# Supplementary material for: Complex‐centric proteome profiling by SEC‐SWATH‐MS
Source: Mol Syst Biol. 2019 Jan 14;15(1):e8438. doi: 10.15252/msb.20188438 (PMC6346213; doi:10.15252/msb.20188438)
Supplement: Supplementary file 6 — Dataset EV5 [file MSB-15-e8438-s006.zip › feature_plots_corum/3070.pdf]

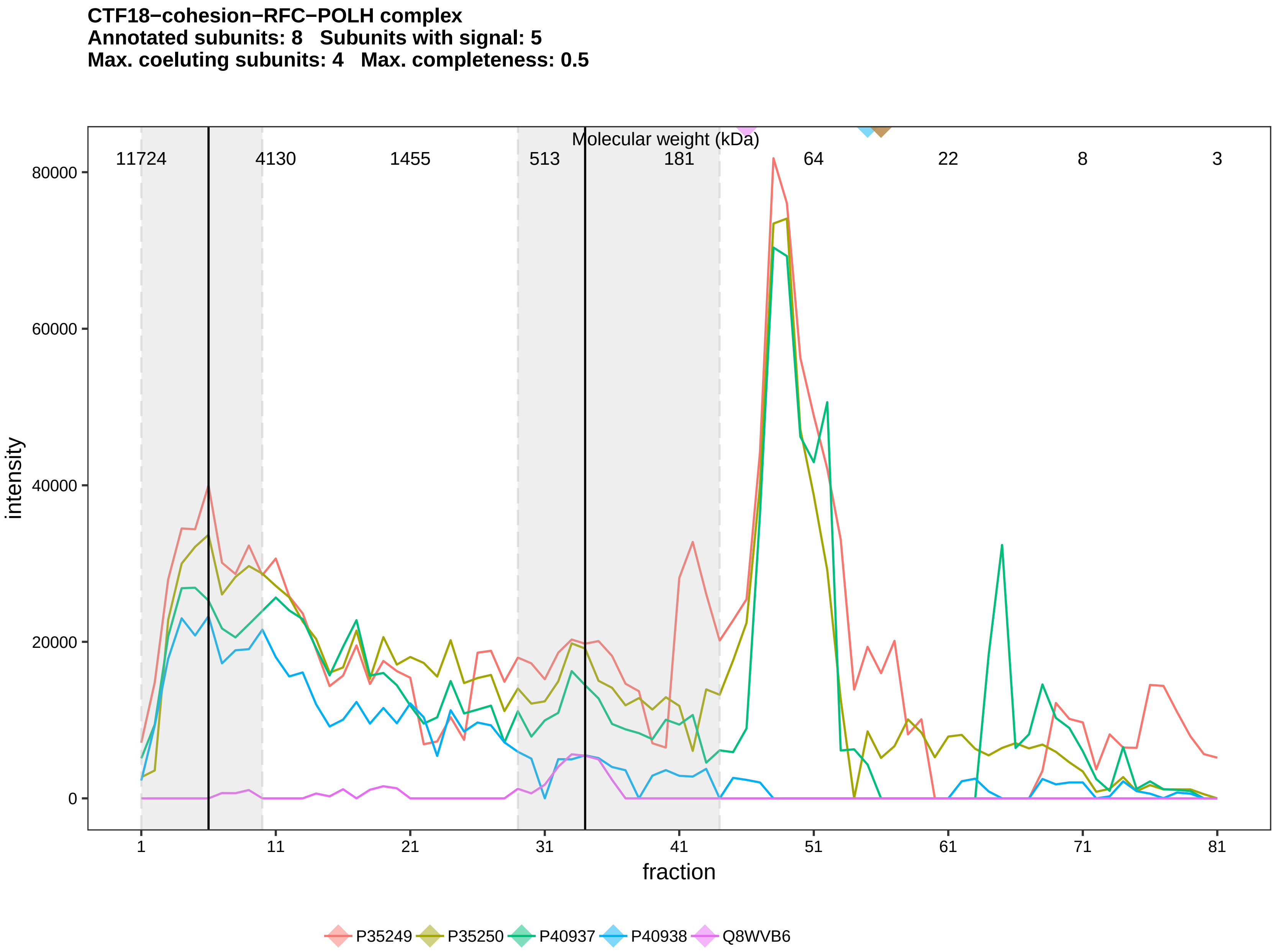

CTF18−cohesion−RFC−POLH complex
Annotated subunits: 8 Subunits with signal: 5
Max. coeluting subunits: 4 Max. completeness: 0.5
Molecular weight (kDa)
11724
4130
1455
513
181
64
22
8
3
intensity
80000
60000
40000
20000
0
1
11
21
31
41
51
61
71
81
fraction
P35249
P35250
P40937
P40938
Q8WVB6
